# Presence of retinal pericyte-reactive autoantibodies in diabetic retinopathy patients

**DOI:** 10.1038/srep20341

**Published:** 2016-02-03

**Authors:** Lingjun Zhang, Yan Li, John Payne, Sunil Srivastava, Xingjun Fan, John Fung, Xiaorong Li, Timothy S. Kern, Feng Lin

**Affiliations:** 1 Tianjin Medical University Eye Hospital, Eye Institute & School of Optometry and Ophthalmology, Tianjin, China; 2 Department of Immunology, Lerner Research Institute, Cleveland Clinic, Cleveland, Ohio, USA; 3 Palmetto Retina Center, West Columbia, South Carolina, USA; 4 Department of Ophthalmology, Cole Eye Institute, Cleveland Clinic, Cleveland, Ohio, USA; 5 Department of Pathology, Case Western Reserve University, Cleveland, Ohio, USA; 6 Digestive Disease Institute, Cleveland Clinic, Cleveland, Ohio, USA; 7 Department of Medicine, Case Western Reserve University, Cleveland, Ohio, USA

## Abstract

The loss of retinal pericytes (RPCs) is a hallmark of early stage diabetic retinopathy (DR), but the mechanism underlying RPC death is unclear. Although it was postulated in previous studies using bovine RPCs that autoantibodies against RPCs might develop and induce RPC death, it is unknown whether autoantibodies against cell-surface antigens on human RPCs exist in DR patients, whether such autoantibodies contribute to RPC damage/loss, and if they do, through which mechanism. We screened serum samples from DR patients and controls using primary human RPCs and found that that levels of IgGs reactive to RPCs were significantly higher in the DR group than the control group. Serum samples with higher RPC-reactive IgG levels induced more severe complement-mediated RPC damage than those with lower RPC-reactive IgG levels. We also assessed levels of the complement-activation products C3a, C4a and C5a in these serum samples, and found that serum levels of C3a and C5a, but not C4a, were higher in the DR group than control group. These data provide evidence the first time showing that autoantibodies against RPCs can develop in DR patients, and that these autoantibodies could contribute to pericyte damage through complement activation.

Diabetic retinopathy (DR) is one of the most common causes of blindness. DR is believed to be a metabolic disease, but the potential involvement of the immune system in DR development and progression has been largely neglected. Recently, increased attention is being paid to the role of inflammation and inflammatory cells in the pathogenesis of DR^1^,^2^,^3^,^4^,^5^. In retina/vitreous of patients/animals with retinopathy, levels of inflammatory cytokines (*e.g.,* IFN-γ and TNF-α) are increased^6^,^7^, levels of adhesion molecules (*e.g*. ICAM-1) which facilitate leukocytes attachment and infiltration are increased^8^, and activated monocytes and granulocytes are identified^9^. Interestingly, reports in the 1960 s suggested that high doses of salicylates (anti-inflammatory doses) inhibited DR^10^, and the ability of corticosteroids to inhibit diabetic macula edema provides further support for the concept that the immune system is integrally involved in the pathogenesis of DR^5^.

Pericytes are critical for maintaining vessel stability and controlling endothelial cell proliferation^11^. Retina has the most abundant pericyte density in the entire body^12^. It is generally considered that the loss of RPC is a hallmark of early stage DR in patients and animal studies, and contributes to the development and progress of DR^13^,^14^. Indeed, while mice are completely lacking pericytes die due to microvascular leakage and haemorrhage^15^,^16^, mice with reduced numbers of pericytes develop microvascular lesions consistent with aspects of DR^17^. Previous *in vitro* studies have shown that RPC die in diabetic animals through metabolic abnormalities-associated mechanisms including oxidative stress^18^, formation of advanced glycation end products (AGEs)^19^, and upregulation of protein C^20^, however, the exact causes of RPC injury and/or death *in vivo* in DR remain elusive, and a potential role of autoimmunity in this process remains unclear.

Complement is an important part of innate immunity, and is abundant in the sera (C3 concentration is ~ 1.2 mg/ml in human sera). It serves as a first shield against invading pathogens by assembling membrane attack complexes (MACs, C5b-9) to directly injure/lyse invading cells, and by releasing anaphylatoxins (i.e. C3a, C4a and C5a) to recruit/activate leukocytes to the site of complement activation^21^. Complement also functions as an effector mechanism for the humoral adaptive immunity. In fact, complement is integrally involved in many autoimmune diseases in which autoantibodies are present, including antiphospholipid syndrome^22^, membranous glomerulonephritis^23^, and myasthenia gravis (MG)^24^,^25^. In these diseases, autoantibodies bind to cell surface antigens on target cells, activate complement, leading to cell injury, tissue destruction and organ dysfunction.

Using primary human RPCs and model antigen-reactive antibodies, including an anti-human leukocyte antigen and an anti-CD38 monoclonal antibody (mAb), we previously provided proof-of-concept that after antibodies bind to the surface of RPCs, complement is activated, which attacks the cells, leading to cellular injury and functional impairment^26^. However, whether or not DR patients develop IgGs against antigens on the surface of RPCs *in vivo* is not clear.

In this study, we analyzed serum samples from 44 non-diabetic controls and 41 DR patients (Table 1 and Supplement) for levels of antibodies reactive to surface antigen(s) on primary human RPCs cultured under hyperglycemic conditions. We then compared the serum samples containing higher levels of RPC-reactive antibodies with those containing lower levels of reactive antibodies regarding their capabilities to injure cultured RPCs via complement activation. We also measured levels of complement activation products, C3a, C4a and C5a, in the serum samples. Our results suggest that autoantibodies against RPCs exist in DR patients, and that these autoantibodies could cause RPC injury/loss in DR patients by activating complement.

## Results

### RPC cell surface antigen-reactive IgG levels are significantly higher in sera from DR patients than in sera from non-diabetic controls

**Figure 1:**
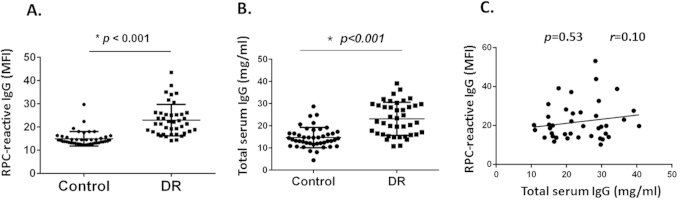
Detection of human RPC surface reactive IgGs in DR patient and non-dabetic controls. (**A**). Serum/plasma samples were diluted and incubated with RPCs cultured in complete media containing 25 mM glucose, then cell surface binding human IgGs were detected by an anti-human IgG secondary antibody and quantitated by flow cytrometric analysis. These experiments were repeated 3 times, and the averaged levels of IgGs (MFI) on RPC from each patient were presented. Each dot represents one patient. *p < 0.001 (**B**). the same samples were also used to measure total IgG levels within them by a conventional ELISA, showing that total IgG levels in the DR group are higher than those in the control group (23.14 ± 1.17 mg/ml vs. 14.69 ± 0.69 mg/ml, *p* < 0.001); (**C**). Spearman’s rank correlation test showed that elevated total IgG levels do not correlate with increased RPC-reactive IgG levels in the DR group (*p* = 0.53, r = 0.10).

We incubated primary human RPCs cultured under high glucose (25 mM) conditions with diluted patient sera, then measured the cell surface bound IgG levels by flow cytometric analysis. We repeated the analysis 3 times to ensure the reproducibility of the data. We found that levels of IgGs bound to cell surface were significantly higher in the DR group than the control group (Fig. 1A) [mean fluorescence intensity (MFI): 22.7 ± 1.0 *v.s.*15.0 ± 0.5, *p* < 0.001], suggesting that autoantibodies against RPC surface antigen(s) develop in DR patients.

Previous studies suggest that total serum IgGs are elevated in diabetes patients^27^,^28^, so there is a slight chance that the increased levels of RPC-reactive IgGs in DR patients might just reflect increased total IgGs in the serum. To exclude this potential pitfall, we measured total serum IgG concentration in both the DR and control groups by ELISA, and found that total IgG levels were indeed higher in the DR group (than those in the control group (23.14 ± 1.17 mg/ml vs. 14.69 ± 0.69 mg/ml, *p* < 0.001) (Fig. 1B). We then did a correlation analysis of all the samples in the DR group to see if increased RPC-reactive IgG levels correlated with elevated total IgG concetrations in the same samples. These statistical analyses showed that there was no statistically signficant correlation between the RPC-reactive IgG levels and total serum IgG concentrations (*p* = 0.53, r = 0.10) (Fig. 1C), demonstrating that the increased RPC-reactive IgG levels in the DR group is not the result of elevated total serum IgG levels in these samples.

### Serum samples containing higher levels of RPC-reactive IgGs induce more complement-mediated RPC damage than do serum samples containing lower levels of RPC-reactive IgGs

**Figure 2:**
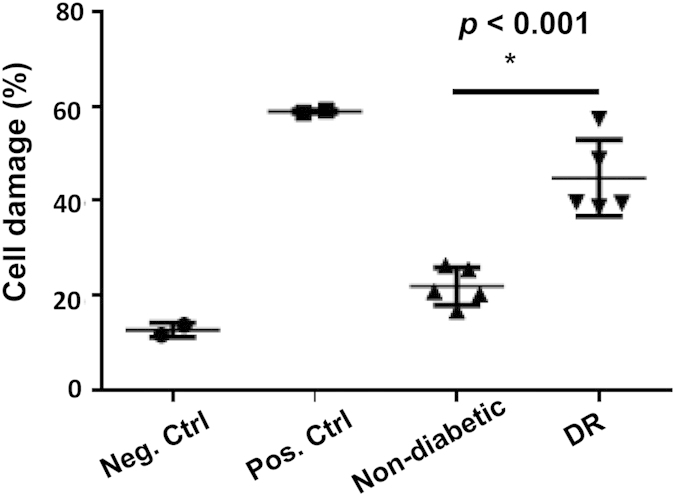
DR patient sera induced complement - mediated RPC damage. 5 serum samples with higher levels of RPC-reactive IgGs were picked from the DR patient group and 5 with lower levels of RPC-reactive IgGs were picked from the healthy control group, and their capability of inducing complement-mediated RPC damage was measured using a BCECF leakage-based cell injury assay. RPCs without any sensitization were included as a negative control (Neg. Ctrl) and RPCs senstitized with anti-HLA ABC IgGs were used as a positive control (Pos. Ctrl). Each dot represents one patient except for the unsensitized and sensitized RPC controls, which represent results from duplicated wells. Data were mean ± SD.

After confirming that the elevated levels of RPC-reactive IgGs in DR group is not an artifact, we next tested the significance of these higher levels of RPC-reactive IgGs. In brief, we picked 5 serum samples from the DR group and 5 from the healthy control group in which the RPC-reactive IgG levels were higher in the DR group than the healthy control group (MFI 24.5 ± 2.7 v.s. 14.8 ± 1.1), then assessed their capacities to induce complement-mediated RPC damage. We also included unsensitized RPCs and anti-HLA ABC IgG-sensitized RPCs as negative and positive controls^26^. These experiment showed that the serum samples from DR patients which contain higher levels of RPC-reactive IgGs induced significantly more complement-mediated RPC damage than did serum samples from controls containing lower levels of RPC-binding IgGs ( cell damage 44.8% ± 7.9% v.s. 22.0% ± 4.0%, *p* < 0.05) (Fig. 2), suggesting that these RPC-reactive autoantibodies can damage RPCs by activating complement.

### The RPC-reactive IgGs in sera do not strongly bind to AGEs

**Figure 3:**
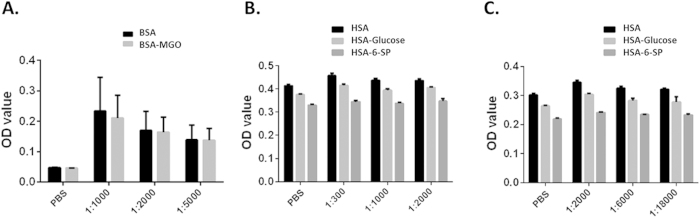
Measurements of AGE-specific IgGs in serum samples by ELISA. Different dilutions of the same serum samples in the DR patient group used in the above complement-mediated RPC damage assay were also analyzed for the presence of AGE-specific IgGs by ELISA using BSA-MGO, HSA-glucose or HSA-6-SP coated plates. No apparent AGE-specific IgGs were detected in plates coated with BSA-MGO **(A)**, or in plates coated with HAS-glucose or HAS-6-SP (**B,C**). (**B**) 1;300-1:2000 serum dilution; (**C**) 1:2000 to 1: 18000 serum dilution). Data were mean ± SD.

Because RPCs in DR patients are exposed to chronic hyperglycemic conditions, and RPCs used in our assays were also cultured in media containing high concentrations of glucose, we speculated that some of the detected IgGs might be reactive against AGEs formed under chronic hyperglycemic conditions. To test this hypothesis, we measured the ability of these IgGs from the serum samples described above to bind to non-enzymatically glycated albumins (HSA-Glucose, HSA-6-SP or BSA-MGO)^29^,^30^,^31^. We found that, in BSA-MGO ELISA experiments, there was no difference of optical density (OD) readings between cells coated with BSA-MGO or BSA alone at various serum dilutions (Fig. 3A). Likewise, in ELISA experiments using plates coated with comparable concentrations of HSA-Glucose, HSA-6-SP or the control HSA, OD readings were even higher in wells coated with control HSA after incubating with anti-human IgG secondary antibodies than those in wells coated with the glycated HSA (Fig. 3B,C). These results suggest a minimal binding of IgGs in the DR patient sera to AGEs, and a potential contamination of human IgGs during the HSA preparation from the plasma/serum. These assays showed that there was no apparent detectable AGE-binding IgGs in serum samples from the DR patients or healthy controls, suggesting that AGEs are not major antigens on RPCs in DR patients.

### Serum levels of complement activation products C3a and C5a, but not C4a, are higher in the DR group than the control group

**Figure 4:**
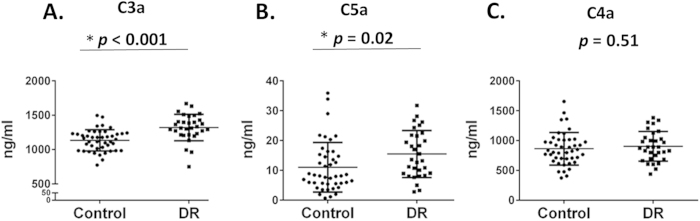
Measurement of complement activation products C3a, C4a and C5a in the serum samples by a BCA assay. Levels of C3a (**A**) and C5a (**B**), but not C4a (**C**) were significantly higher in the DR group than the healthy controls. Data were mean ± SD. Each dot represents one patient.

After antibodies bind to antigens, complement activation will be initiated. Levels of the released complement activation products, i.e., C3a, C4a and C5a, are frequently used to assess the magnitude of complement activation *in vivo*. We next measured levels of these complement activation products in all the serum samples using a cytometric beads assay. These assays (Fig. 4) showed that serum levels of C3a and C5a were significantly higher in the DR group than in the controls (1321.3 ± 188.3 ng/ml v.s.1135 ± 154.3 ng/ml for C3a, *p* < 0.001; and 15.4 ± 8.0 ng/ml v.s.11.0 ± 8.3 ng/ml for C5a, *p* = 0.02), whereas serum levels of C4a did not significantly differ between the two groups (902.0 ± 239.3 v.s.863.8 ± 273.4 ng/ml, *p* = 0.52).

### Correlations among RPC-reactive IgG levels, complement activation and other clinical characteristics in the DR patients

**Figure 5:**
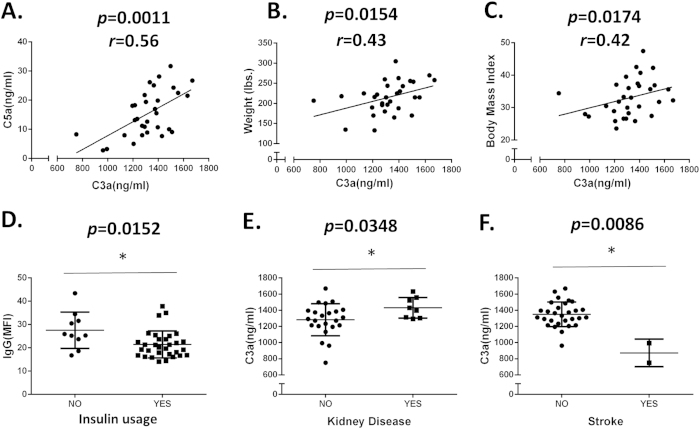
Correlation analyses between levels of RPC-reactive IgGs, C3a and C5a and DR patient clinical characteristics. These analyses showed significant correlation between levels of C3a and C5a (**A**), C3a and weight (**B**), C3a and body weight index (**C**), RPC-reactive IgGs and insulin usage (**D**), C3a and presence of kidney diseases (**E**), and C3a and stroke (**F**). Significance of correlation was analyzed by Spearman’s rank correlation test in panels (**A–C**) and by Mann-Whitney test in panels (**D–F**).

We also did correlation analyes among RPC-reactive IgG levels, complement activation products levels and other clinical characteristics in the DR patients such as weight, body mass index, insulin usage, renal problems and stroke. We found that levels of C3a and C5a were positively correlated (Fig. 5A, *p* = 0.0011), while levels of C3a were also positively correlated with weight (Fig. 5B, *p* = 0.0154) and body weight index (Fig. 5C, *p* = 0.0174). These analyses also showed that RPC-reactive IgG levels were lower in DR patients who used insulin than those who did not (Fig. 5D, *p* = 0.0152), that serum C3a levels were elevated in DR patients who had renal problems (Fig. 5E, *p* = 0.0348) and reduced in the sera of the two DR patients who had stroke (Fig. 5F, *p* = 0.0086).

## Discussion

Using primary human RPCs, we have screened serum/plasma samples from DR patients and non-diabetic controls for the presence of RPC-surface antigen-reactive autoantibodies. We also tested the significance of these RPC surface antigen-reactive autoantibodies in inducing complement-mediated RPC damage, and explored the potential targets of these autoantibodies. Finally, we assessed complement activation levels in DR patients and controls by measuring levels of complement activation products C3a, C4a and C5a in the serum samples. We found evidence suggesting that autoantibodies against RPC cell surface antigen (s) develop in DR patients, and these autoantibodies induce complement-mediated RPC damage *in vitro*. In addition, levels of complement activation products C3a and C5a, but not C4a are higher in the DR group than the control group, suggesting a potential role of the RPC-reactive autoantibodies in activating complement to damage RPCs.

The presence of RPC-reactive autoantibodies in DR patients has been sought previously, but never confirmed. One group reported more than a decade ago that antibodies reacative against *bovine* RPC existed in some diabetic patients as analyzed by indirect immunofluorescence microscopy^32^,^33^. This approach, however, has potential problems: 1) it is well known that most humans developed antibodies against non-human antigens such as Gal alpha-(1,3) Gal^34^, which would react to these xenoantigens on bovine RPCs, resulting in false positive results; 2) protein sequences are different between human and bovine species, which could result in false negative results if the anti-human RPC antigen antibodies fail to recognize the bovine counterparts, and 3) the sensitivity and quantification of the immunofluorescence microscopy assay could be issues too. Subsequently, research in this area languished and the presence or absence of antibodies against pericytes has not been confirmed by any other group(s). Using primary human RPCs in this report, we demonstrated evidence the first time that autoantibodies against human RPC surface antigen(s) develop in DR patients.

Although our flow cytometric analysis suggests that RPC surface-reactive autoantibodies developed in the DR patients tested, and our *in vitro* RPC cytotoxicity assays provide evidence that these autoantibodies were capable of inducing complement-mediated RPC injury, it is still not clear whether these autoantibodies were a cause of RPC death observed in DR development, or whether these antibodies were a result of RPC death (because of the death of RPC exposes the previously sequestered antigens on RPCs to the immune system in DR patients). Moreover, it is not clear whether these damages are specific in DR, or happens in all patients with diabeties. Additional studies are needed using serum samples from diabetic patients prior to DR development with a continuous follow-up to address these issues.

The nature of the RPC-reactive IgGs that developed in DR patients also needs to be further characterized. Previous studies showed that AGEs formed on cell surface under hyperglycemic conditions could be recognized by the immune system and serve as neoantigens to elicit antibody production^29^,^30^,^31^. Our ELISA results, however, do not support the hypothesis that AGEs on RPCs are the primary neoautoantigens that antibodies in DR patient recognize, because we did not detect appreciable levels of antibodies that bind to albumin modified by glycating metabolites that are characteristic of diabetes. These negative results suggest that proteins on RPC surface in DR patients are likely to be the antigens that these antibodies recognize.

A few reports support a role of complement in the development of DR: 1) MAC deposition has been observed within retinal blood vessels of diabetic patients^35^,^36^, 2) C3 and complement factor I were found increased in vitreous of patients with proliferative DR^37^, and 3) Immunohistological study of pre-retinal membranes from diabetic patients showed deposition of complement components within the connective stroma and along new vessels^38^, as well as the presence of complement activation products C3d, MAC, and vitronectin in the choriocapillaris of eyes with DR. Our detection of RPC-reactive autoantibodies and the demonstration that these autoantibodies induced complement-mediated RPC damage *in vitro* raise the possibility that these RPC-reactive autoantibodies could contribute to RPC damage/loss *in vivo* in DR patients. Interestingly, we found that serum levels of C3a and C5a, but not C4a, were elevated in DR patients compared with controls. The failure of C4a to increase is not surprising, as C4a is only generated when the classical or lectin pathway of complement activation is initiated, whereas both C3a and C5a, products of the activation of C3 and C5 can be generated in all complement activation pathways, both at the initiation phase and at the amplification phase. The modest increase of C3a and C5a levels in the serum samples from DR patients could due to levels of C3a and C5a that we detected in the sera reflected systemic, but not local (retinal) levels, and the local (retinal) levels of C3a and C5a could be significantly higher.

RPC-reactive IgGs are lower in DR patients who are using insulin than those who do not, suggesting that insulin usage could directly influence RPC-reactive autoantibody production in patients. It is not surprising that C3a levels are positively correlated with C5a levels in the examined DR patients, as when complement is activated, both C3a and C5a are produced. It is interesting to see that C3a levels are also positively correlated with weight, and weight mass index, suggesting that obesity could lead to elevated complement activation, or *vice versa*.

In summary, using primary human RPCs cultured under hyperglycemic conditions, we found evidence that autoantibodies against human RPC cell surface antigens exist in sera of DR patients. These autoantibodies induced complement-mediated RPC damage *in vitro*. In DR patients, breakdown of the retinal-blood barrier allows immunoglobins and complement in the blood to have access to RPCs^5^. We hypothesize that autoantibodies against cell surface antigens on RPC bind to RPC surface and activate complement, then activated complement can contribute to RPC cytotoxicity, dysfunction, and eventually, cell death.

## Methods

### Study subjects

Serum/plasma samples were collected from age- and sex-matched controls (no diabetes mellitus), and type 2 diabetes patients diagnosed with proliferative DR at Emory Eye Center, Emory University between December 16, 2009 and March 21, 2010. Informed consent was obtained form all subjects. Subjects were excluded if they were younger than 18, or were older than 90 years of age. Patients who were cognitively impaired or unable to provide written informed consent were also excluded. The patient sample collection procedure was approved by the Institutional Review Board (IRB) at the Emory University, and the samples were studied in accorfanced with the approved guidelines. The de-identified patient information can be found in the Supplement.

### RPC culture

Primary human RPCs isolated from a healthy donor (passage 3) were purchased from ANGIO-PROTEMIE (Boston, MA) and cultured in Dulbecco’s modified Eagle’s medium (DMEM) with 10% fetal bovine serum ( FBS; Invitrogen, Carlsbad, CA). RPCs were maintained in complete DMEM media with high concentration of glucose (25 mM) for at least 14 days. Culture media was changed every other day. RPCs on passage 6 were used in all the experiments.

### RPC- reactive antibody level measurements by flow cytometry

RPCs were harvested and incubated in DMEM media with 30% serum/plasma samples of healthy controls or DR patients for 1 h at 37 °C. After washing, autoantibodies on RPCs were detected by incubating the cells with 5ug/ml of Alexa Fluor® 488-conjugated donkey-anti-human IgG (Jackson Immunoresearch Laboratories Inc., West Grove, PA) for 30 min on ice. RPCs were then washed and analyzed by flow cytometry. Data collection was performed on a FACS Calibur flow cytometer (BD Biosciences, San Jose, CA), and analyzed using FlowJo (Version 7.6.5). Measurments were repeated 3 times.

### Total serum IgG concentration measurements

The total serum IgG levels of control and DR were measured by ELISA. Briefly, Goat anti-Human IgG capture antibody (Jackson ImmunoResearch, West Grove, PA) was diluted to 10 μg/ml with PBS and 100 μl was added to each of the wells of a microtiter plate. After overnight incubation, sites unoccupied by antibody were blocked by 1% BSA in PBS. After washing, 100 μl human serum samples with 1 to 1 × 10^5^ dilution were incubated at room temperature for 2 h. Then 100 μl of 1.5 μg/ml Mouse anti-Human IgG conjugated to Alkalin Phosphatase (Jackson ImmunoResearch, PA) was applied to detect the IgG binding to the plate. The p-nitrophenyl phosphate (PNPP) substrate (Thermo scientific, Rockford, IL) was added after washing and the absorbance was measured at 405 nm using a plate reader (SpectraMax Gemini XS, Molecular Devices, Sunnyvale, CA).

### Complement-mediated RPC damage assay

To assess the capability of the IgGs in the serum samples to induce RPC damage, 5 serum samples with higher levels of RPC-reactive IgGs in the DR group and 5 serum samples containing lower levels of RPC-binding IgGs in healthy controls group were selected and tested in a previously established complement-mediated cell damage assay based on measuring the leakage of the fluorescence dye, 2′,7′-bis-(2-carboxyethyl)-5-(and-6)-carboxyfluorescein (BCECF)^26^,^39^,^40^. In brief, 2 × 10^5^ RPCs were incubated with 5 μM of 2′,7′-bis-(2-carboxyethyl)-5-(and-6)-carboxyfluorescein acetoxymethyl ester (BCECF-AM) (Thermo Fisher Scientific™, Waltham, MA) for 30 min at 37 °C. After washing, the BCECF-loaded RPCs were incubated with 5 μg/ml of anti- human leukocyte antigen (HLA) ABC IgG (Clone W6/32, Thermo Fisher Scientific™, Waltham, MA) (positive control), or 30% patients’ sera in 100 μL of gelatin veronal buffer (GVB^**o**^) (10 mM Barbital, 5 mM Veronal, 145 mM NaCl, 0.1% Gelatin, pH 7.2, CompTech, Tyler, TX) for 30 minutes on ice to allow IgGs bind to cell surface without complement activation. After this, the IgG-sensitized RPCs were washed and re-suspended in 100 μL of GVB +  + (GVB^**o**^ plus 0.5 mM MgCl_2_, 0.15 mM CaCl_2_) containing 30% of pooled normal human serum (CompTech, Tyler, TX) as a source of complement. After 30 min incubation at 37 °C, the RPCs were spun down and then supernatant was collected to measure levels of BCECF leaked from complement-damaged RPCs by SpectraMax Gemini XS (Molecular Devices, Sunnyvale, CA) with excitation and emission wavelengths of 485 nm and 538 nm. The percentage of BCECF release due to complement-mediated cell damage was calculated, the percentage of BCECF release = ([A − B]/[C − B]) × 100%, where A represents the mean experimental BCECF release, B represents the mean spontaneous BCECF release (when complement activation is inhibited by 1 mM EDTA), and C represents the mean maximum BCECF released that was induced by incubating cells with 0.1% Triton-x100.

### AGE-reactive IgG ELISA

To assess whether or not the RPC-reactive IgGs recognize AGEs, the same DR patient serum samples described above were analyzed for AGE-specific IgGs by ELISA. Human serum albumin (HSA), AGE-HSA-DG (HSA incubated with 100 mM D-glucose for 6 weeks), AGE-HSA-6 P (HSA incubated with 100 mM glucose-6-phosphate for 6 weeks), bovine serum albumin (BSA), or MGO-BSA (BSA incubated with 100 mM of methylglyoxal (MGO) for 7 days) were used at 5ug/ml to coat the wells of an enzyme immunoassay plate (Greiner bio-one, Monroe, NC). Sera diluted in various concentrations (1:300~1:18000) were incubated and AGE-reactive IgG was detected using 2ug/ml biotin anti-human IgG (BioLegend, San Diego, CA) and alkaline phosphatase anti-biotin (Vector Laboratories Inc., Burlingame, CA) antibodies. Substrate PNPP was added and the optical density at 405 nm was measured after color development.

### Complement anaphylatoxins measurements

Levels of complement anaphylatoxins, including C3a, C4a and C5a in all the serum samples (44 in control group and 31 in DR patient group) were measured by Cytometric Bead Array (CBA) Kit (BD Biosciences, San Jose, CA) according to the manufacturer’s instructions. Briefly, 50uL of serum samples with 1:1500 dilution or known concentrations of anaphylatoxins standards were added to a 50ul mixture of each capture beads. The samples were incubated for 2 h at room temperature in the dark. After washing, all assay samples were incubated with 50ul of phycoerythrin (PE)-conjugated detection antibody for 1 h and then washed to remove the unbound antibody. Data were acquired using a FACS Calibur flow cytometer, and analyzed by FCAP Array^TM^ Software (Version 3.0, BD Biosciences).

### Statistics

Data were expressed as individual values and means ± SD. The differences between groups were compared by Student’s *t* test.The correlation analyses were performed by software JMP9.0, and the correlations between levels of total IgG, RPC-reactive IgG, C3a, C5a and other clinical characteristics of DR patients were tested by Spearman’s rank correlations or Mann-Whitney test. A liner regression analysis was preformed to compare correlations among the relevant parameters. A *p* value less than 0.05 was considered statistically significant.

## Additional Information

**How to cite this article**: Zhang, L. *et al*. Presence of retinal pericyte-reactive autoantibodies in diabetic retinopathy patients. *Sci. Rep.* **6**, 20341; doi: 10.1038/srep20341 (2016).

## Supplementary Material

Supplementary Information

## Figures and Tables

**Table 1:** Characteristics of study participants.

	Control (n = 44)	DR patients (n = 41)
Age at Exam	62.13 ± 11.59	59.90 ± 12.13
Race	Black = 23 White = 20 Other = 1	Black = 23 White = 17 Other = 1
Sex	Male = 25 Female = 19	Male = 21 Female = 20
Height (in.)	67.45 ± 3.79	67.29 ± 4.03
Weight (lbs.)	183.02 ± 40.47	213.66 ± 41.54
Body Mass Index	28.23 ± 5.89	33.25 ± 6.62
Years of Diabetes	0	22.12 ± 10.74
Insulin	No = 44 Yes = 0	No = 10 Yes = 31
Hypertension	No = 14 Yes = 30	No = 3 Yes = 38
Number of BP meds	1.63 ± 1.52	3.10 ± 1.95
ACE Inhibitor/ARB	No = 20 Yes = 22	No = 9 Yes = 32
Aspirin	No = 29 Yes = 15	No = 23 Yes = 18
Cholesterol	No = 20 Yes = 22	No = 12 Yes = 19
Statin	No = 28 Yes = 16	No = 26 Yes = 15
Heart Attack	No = 39 Yes = 5	No = 30 Yes = 11
Stent or CABG	No = 40 Yes = 4	No = 28 Yes = 13
Stroke	No = 42 Yes = 2	No = 36 Yes = 5
Kidney Disease	No = 44 Yes = 0	No = 30 Yes = 11
HbA1c level (%)	5.81 ± 0.5	8.23 ± 1.80
IGF-1 Level (ng/ml)	76.74 ± 27.82	91.885 ± 60.26
Serum Creatinine (mg/dl)	0.95 ± 0.21	2.55 ± 2.84
Estimated GFR (ml/min)	81.95 ± 20.32	47.71 ± 30.23
